# Comprehensive evaluation and application of woody plants in the green spaces of parks in saline–Alkaline areas from a low-carbon perspective: A case study of Tianjin Qiaoyuan Park

**DOI:** 10.1371/journal.pone.0303341

**Published:** 2024-05-10

**Authors:** Jieyuan Bai, Hongcheng Wang

**Affiliations:** College of Architecture, Tianjin University, Tianjin, 300072, China; BOKU: Universitat fur Bodenkultur Wien, AUSTRIA

## Abstract

The field of landscape architecture has placed significant emphasis on low-carbon landscapes due to the increasing challenges posed by global warming and environmental deterioration in recent years. The soil ecological conditions in saline–alkaline areas are characterized by poor quality, resulting in suboptimal growth conditions for trees. This, in turn, hampers their ability to effectively sequester carbon, thereby diminishing the potential benefits of carbon sinks. Additionally, the maintenance of tree landscapes in such areas generates more carbon emissions than does conventional green land, making it difficult to reap the benefits of tree-based carbon. A comprehensive evaluation of trees in green park spaces in saline–alkaline areas is conducted from a low-carbon perspective; by identifying the dominant tree species that are well suited to greening, we can offer a precise scientific foundation for implementing low-carbon greening initiatives in cities situated in saline–alkaline environments. Therefore, as a case study, this study investigates Tianjin Qiaoyuan Park, a typical saline park in the Bohai Bay region. The hierarchical analysis method (AHP) was used to evaluate 50 species of trees and shrubs in the park from a low-carbon perspective. The results show that the evaluation system consists of four criterion layers and 15 indicator factors. The relative weight of the criterion layer followed the order of habitat adaptability (B2) > carbon sequestration capacity (B1) > low-carbon management and conservation (B3) > landscape aesthetics (B4). The indicator layer assigned greater weight values to net assimilation (C1), saline and alkaline adaptability (C3), drought tolerance (C4), irr igation and fertilization needs (C8), growth rate (C2), and adaptability to barrenness (C5). The trees were classified into five distinct categories, with each exhibiting significant variation in terms of the strengths and weaknesses of the indicators. According to the comprehensive score, the trees were categorized into three levels. The Grade I plants exhibited the best carbon efficiency performance, comprising a total of 12 species (e.g. *Sabina chinensis*, *Fraxinus chinensis ’Aurea’ and Hibiscus syriacu*), and demonstrated superior performance in all aspects. Grade II trees, consisting of 26 species (e.g *Pinus tabuliformis*, *Paulownia fortunei*, *Ligustrum × vicaryi*), had the second-highest comprehensive score. Moreover, Grade III trees, encompassing 12 species (e.g *Acer mono*, *Cedrus deodara*, *Magnolia denudata*), exhibited lower comprehensive scores. The extensive use of Grade I and II tree species is recommended in the implementation of low-carbon greening projects in the Bohai Bay region, while Grade III tree species should be judiciously utilized. The findings of this research can serve as a valuable resource for the scientific identification of tree species that are suitable for urban park green spaces in the Bohai Bay region, which is characterized by predominantly saline and alkaline soil. Additionally, the development of an evaluation system can guide the selection of low-carbon tree species when evaluating other types of saline and alkaline lands.

## 1. Introduction

The ongoing processes of global industrialization and urbanization have led to the expansion of industrial cities, which in turn has led to a significant increase in carbon emissions and environmental pressure. Addressing the issue of climate change and attaining carbon peaking and neutrality have emerged as substantial global undertakings [1]. As the largest developing country in the world, China has pledged to achieve carbon peaking by 2030 and carbon neutrality by 2060 [2]. This has generated new requirements for China’s environmental protection and sustainable development. Globally, approximately 20% of irrigated land is suffering from the negative impacts of salinization [3], and the total area of saline land in China amounts to 99.13 million hm^2^, accounting for 10% of the country’s land area [4]and 25% of its arable land area [5]. Saline and alkaline areas have fragile vegetation ecosystems and poor environmental carrying capacity [6], which are major obstacles to sustainable economic and social–ecological development but also necessarily reserve land resources. The poor structural properties of saline soils, along with their poor permeability, shallow groundwater depth, and infertility [7], make it difficult to choose varieties for landscaping. This reduces the effectiveness of green landscapes and carbon sequestration benefits.

Under normal conditions, the carbon emissions generated by the management of a mature tree annually amount to approximately 2400 g [8]. However, in saline–alkali areas, due to the presence of barren soil, the amount of soil improvement and maintenance work required for landscaping in the later stages—such as soil plowing and desalination [9], fertilization and irrigation [10], and vegetation removal [11]—needs to increase compared to that in conventional green spaces. This will further restrict the sustainable development of urban landscaping in saline‒alkali areas. Studies have shown that areas covered by trees in cities have an average annual carbon storage of approximately 7.69 kg m^-2^ [12], and the changes in soil organic carbon are positively correlated with changes in vegetation cover and productivity [13]. By selecting appropriate tree species, urban greening can not only enhance ecosystem service functions but also significantly improve carbon sequestration capacity, and reduce greenhouse gas emissions, thus contributing to addressing global climate change [14]. In this context, integrating low-carbon concepts into greening saline–alkali areas and choosing greening tree species with high carbon sequestration and low carbon emissions can help accelerate the ecological restoration and sustainable development of greening construction in saline–alkali lands. Considering the unique habitat conditions of saline–alkali lands, there is an urgent need to establish a comprehensive evaluation system for low-carbon trees suitable for saline–alkali conditions to provide a scientific and rational basis for the selection of superior varieties and to promote the sustainable construction of urban greening in saline–alkali areas.

As early as the 1940s, researchers from the former Soviet Union, Hungary, and other regions carried out preliminary research on the selection and breeding of salt-tolerant tree species, saline–alkaline land afforestation technology, secondary salinization, and improvement counter measures [15]. Subsequently, scholars have carried out extensive research on the salt tolerance of trees from the perspective of physiological mechanisms. For example, Sanada et al. determined the salt tolerance of different trees by measuring the accumulation of proline [16]. Hurkman et al. used two-dimensional polyacrylamide gel electrophoresis to analyze the effects of salt stress on polypeptide and mRNA levels in the roots of two different salt-tolerant barley varieties to evaluate their salt tolerance [17]. In recent years, studies on saline tree selection have begun to incorporate factors affecting other tree dimensions rather than just considering tree salt tolerance. For example, Menninge comprehensively considered harsh habitat conditions in coastal saline–alkaline land. He identified nearly 2,000 species of plants with high tolerance to these conditions in his book *Seaside Trees of the World* [18]. Zaurov et al. explored the drought, cold, and salinity tolerance of apricots in Central Asia [19]. Su and He evaluated the landscape of central shrubs in the coastal saline–alkaline land of Hangzhou Bay according to three aspects: the ornamental value, biological characteristics, and growth adaptability of the trees [20]. In summary, studies evaluating trees in saline–alkaline areas have focused primarily on their salt tolerance, stress resistance function, and aesthetic function while ignoring their carbon sequestration capacity and the carbon footprint they produce throughout their whole life cycle. With the intensification of climate change, the habitat conditions of saline–alkaline lands are becoming worse, and the requirements for tree selection are also changing accordingly. Therefore, there is an urgent need for a comprehensive evaluation system for low-carbon trees for the unique habitat and site conditions of saline–alkaline land to provide a scientific and reasonable basis for the selection of dominant species and as a reference for the sustainable construction of urban greening in saline–alkaline areas.

The main comprehensive evaluation methods include fuzzy mathematics [21], gray relational analysis [22], the analytic hierarchy process [23], and principal component analysis [24]. The analytic hierarchy process (AHP) is a method of analysis that combines qualitative and quantitative analysis. Quantitative analysis is conducted after various influencing factors are comprehensively examined through hierarchical division and weight distribution, thereby reducing the subjectivity of qualitative analysis [25]. At present, this method has been widely used in tree evaluation and application research, such as in sponge cities [26], ecological landscapes [27], sustainable landscapes [28], and low-carbon landscape [29] construction.

The Bohai Bay area is undergoing rapid urbanization, and there is a large amount of saline–alkaline land [30]. The selection of suitable tree species has important theoretical and practical significance for promoting the low-carbon development of entire regions. Tianjin Qiaoyuan Park is a typical saline–alkaline park in the Bohai Bay area. A large proportion of the park is green space and is rich in plant resources. It has relatively successful experience in ecological green landscape construction and is suitable as a research object for evaluating green plants in saline–alkaliine land parks. Based on a comprehensive survey of its plants, this approach involves integrating factors affecting plant carbon benefits, saline–alkali soil habitat conditions, and the aesthetic requirements of park greening plants. By employing the AHP, a multidimensional comprehensive evaluation system is established. The objective of the evaluation system is to identify superior composite tree species characterized by high carbon sequestration benefits, ease of maintenance in saline‒alkali environments, and beautiful landscapes.

## 2. Materials and methods

### 2.1. Overview of the study area

**Fig 1:**
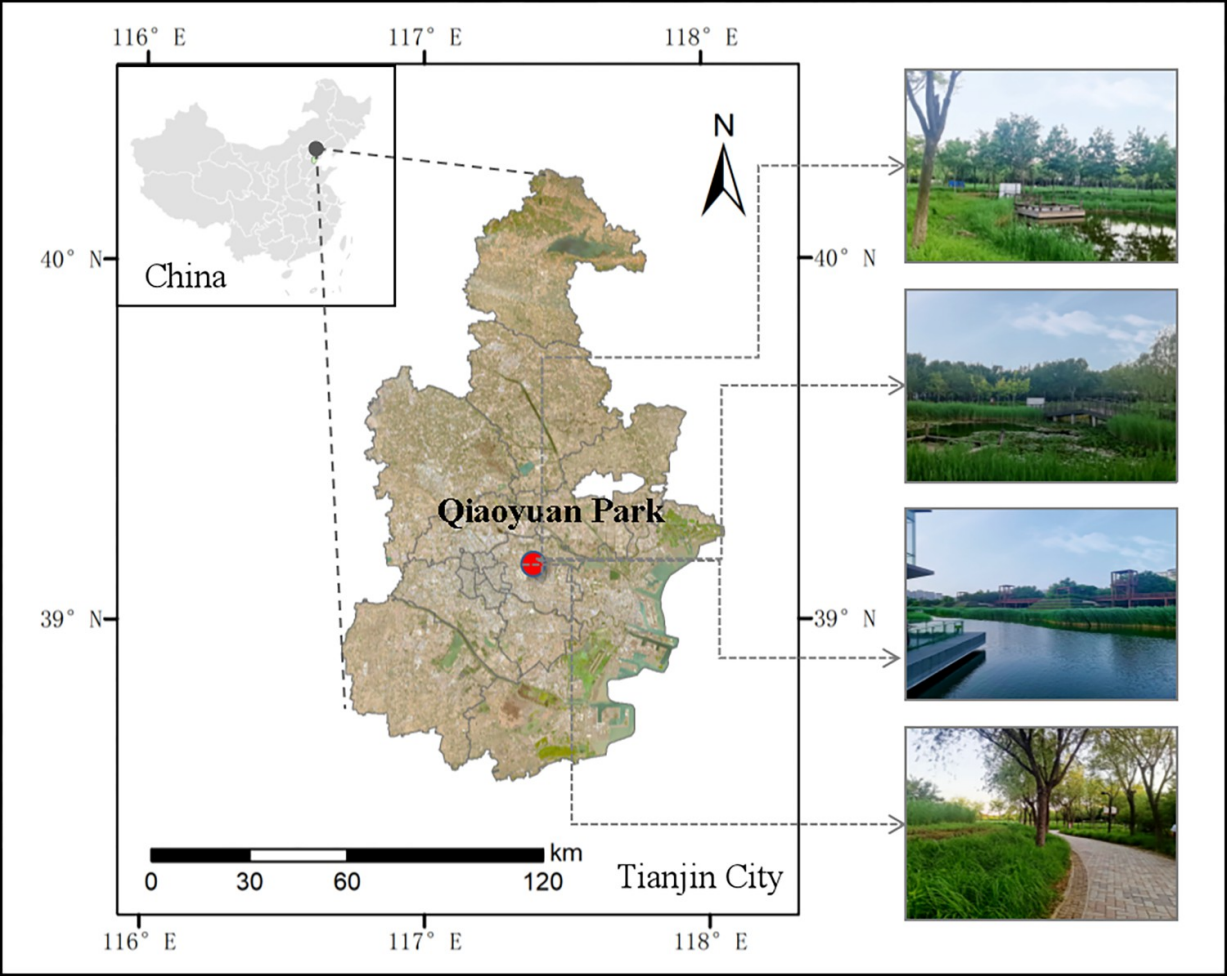
Location of the study area.

As shown in Fig 1, Tianjin is located in northern China, facing the Bohai Sea in the east, and has a coastline of 153.67 kilometers. The salinized land area accounts for approximately 65.80% of the city’s total area, and the degree of salinity varies; moreover, the soil status has limited the selection of greening plant materials [31]. Tianjin Qiaoyuan Park is located in Hedong District, Tianjin city, with a total area of approximately 26.7 hm^2^. The park has ample green space, and the soil pH is between 7.6 and 8.8, which constitutes mild to moderate saline–alkali soil [32]. This is similar to the soil H range of 6.9 to 8.5 found in the green areas around the four districts of Tianjin city [33]. Since the park was designed, there has been a commitment to building an urban park with low maintenance costs and high ecological benefits [34]. Most park plants are resistant to salt, alkalis, water, and humidity, especially native tree species such as *Salix matsudana*, *Fraxinus chinensis*, *Sophora japonica*, *Diospyros kaki*, *Lonicera maackii*, and *Hibiscus syriacus*. The total number of trees and shrubs in the park comprises 24 families, 42 genera, and 50 species, including 4 species of evergreen trees, 34 species of deciduous trees, 1 species of evergreen shrubs, and 11 species of deciduous shrubs. The species composition is rich, and the community structure is diverse, essentially encompassing the tree species commonly used for greening saline–alkali lands in Tianjin [35].

### 2.2. Research framework

**Fig 2:**
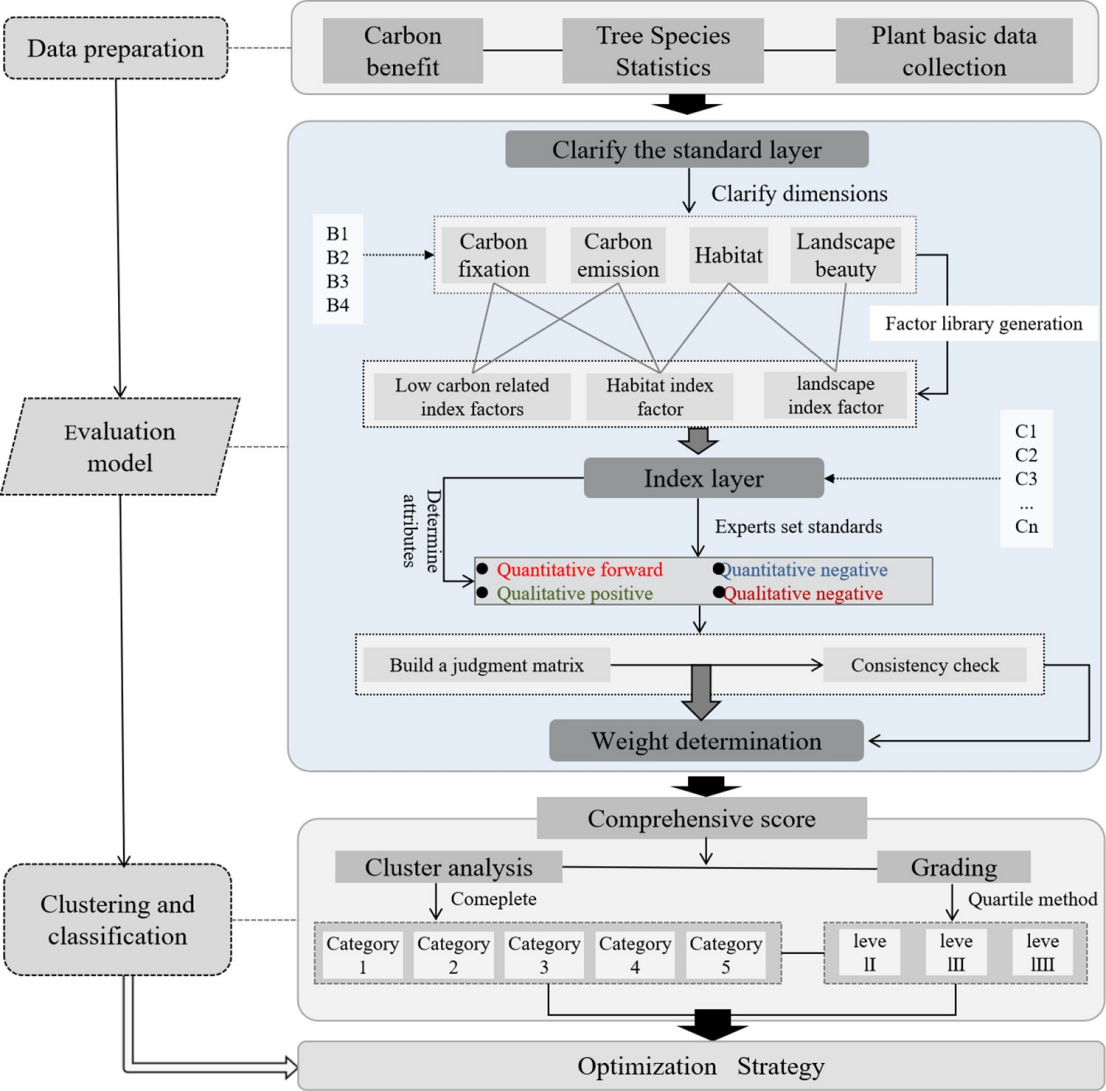
Research framework.

As shown in Fig 2, the research is carried out according to the data preparation, evaluation model, clustering and classification, and application strategies. First, we prepared the data for the study, which included three steps: measuring the carbon benefits of trees, collecting the statistics of the varieties, and collecting the primary data. Second, an evaluation model is constructed, focusing on clarifying the dimensions from which the evaluation criterion layer is considered and defining the evaluation criteria and attributes of the subsidiary indicator layer. The comprehensive weights are obtained through the construction of a judgment matrix and a consistency test. Third, after the comprehensive scores are assigned, the indicator scores are clustered using the complete systematic clustering method, and the comprehensive scores are hierarchically classified via the quartile method. Finally, based on the results, an optimization strategy for plant application is proposed.

### 2.3. Data sources

#### 2.3.1. Collection of plant carbon sequestration data

**Fig 3:**
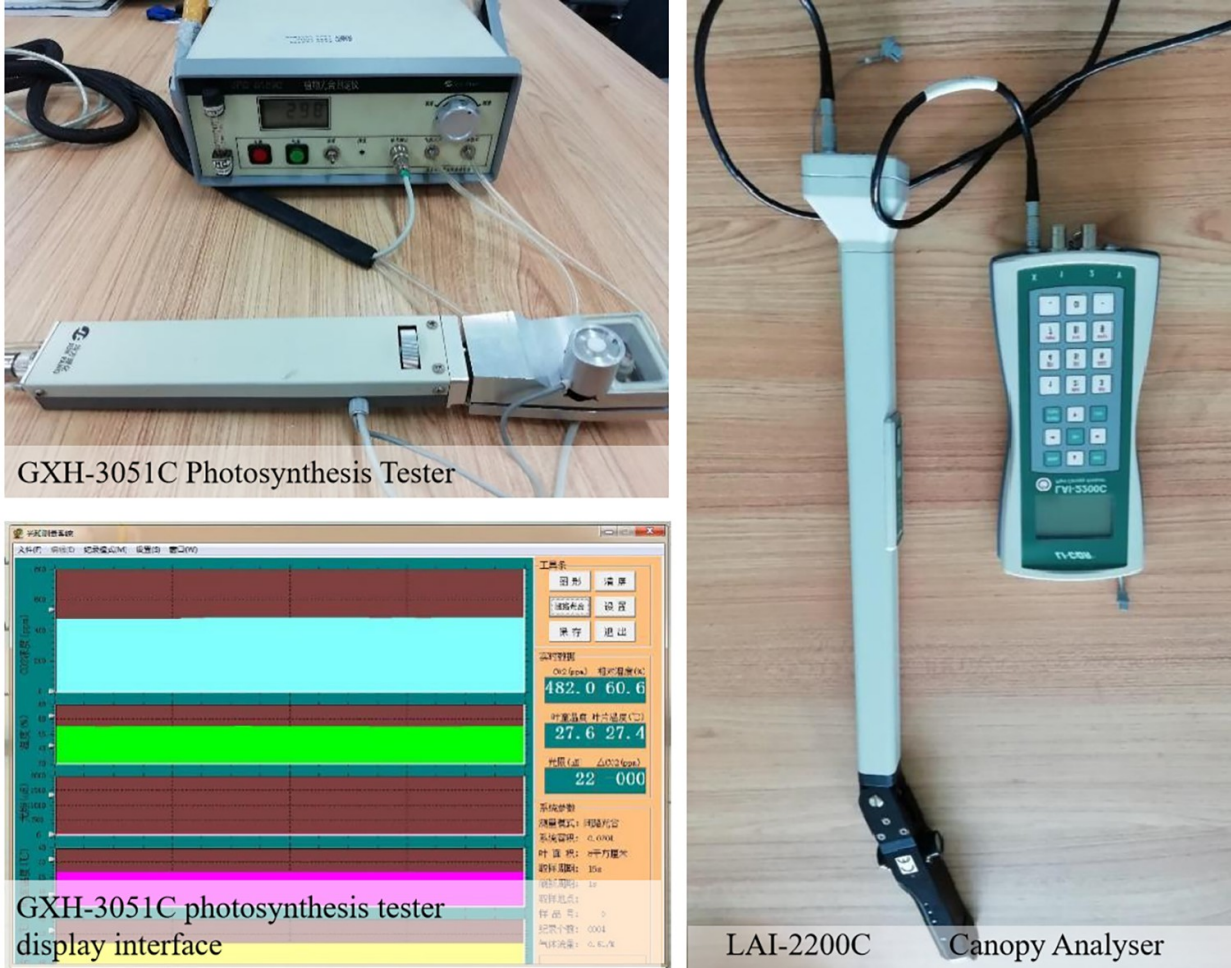
Instruments required for data collection related to the carbon benefits of plants.

The assimilation method is used to obtain the instantaneous photosynthetic rate and respiration rate per unit leaf area of the plant by instantaneously measuring the *CO*_2_ concentration and *H*_2_*O* in and out of the plant leaves; then, the plant leaf area is multiplied by the net photosynthetic amount of the plant per unit time to obtain the carbon fixation of the plant [36]. The assimilation method has the advantages of allowing nondestructive real-time monitoring, and it achieves high precision. It is often used to evaluate the carbon sequestration capacity of different plants on a small scale and to screen for high-carbon-sink species [37]. More than five healthy plants of the same species with similar ages and diameter classes were selected from the Tianjin Qiaoyuan Park, and the data collection was completed from June 1 to September 1, 2022. The summer in Tianjin (June to September) is warm and humid, which is the most active period for the growth of most woody plants [38]. Therefore, selecting this time frame can more accurately reflect the carbon sequestration efficiency of trees during their peak growth period.Three leaves in the middle of the sunny side of each tree were randomly selected, and five instantaneous photosynthetic rate values were taken for each leaf as a repetition. To reduce the error of the experimental data, the data were collected in clear and cloudless weather, and based on the regularity of plant photosynthesis during the day, the period of 07:00–19:00 was selected for the use of the GXH-3051C photosynthetic measuring instrument, with measurements conducted once every two hours [39]. The leaf area index was measured using an LAI-2200C7 canopy analyzer (Fig 3), and the average score was used to determine the photosynthetic rate and leaf area index. Assuming that the net assimilation amount is p, formula 30 for the net assimilation amount of various plants on the day of measurement [40] is as follows:

$$P=\sum_{i=1}^{j}\left[(p_{i+1}+p_{i})÷2\times (t_{i+1}-t_{i})\times 3600÷1000\right]$$ (1)

where *P* is the total assimilated amount on the day of determination (mmol·m^2^·d^-1^); *p*_*i*_ is the instantaneous photosynthesis rate at the initial measurement point (μmol·m^-2^·s^-1^); *p*_*i*+1_ is the instantaneous photosynthesis rate at the next measuring point (μmol·m^-2^·s^-1^); *t*_*i*_ is the instantaneous time of the initial measurement point (h); *t*_*i*+1_ is the time of the next measuring point (h); and *j* is the testing frequency.

According to the photosynthesis reaction equation ${CO}_{2}+4H_{2}O\to CH_{2}O+3H_{2}O+O_{2}$, the daily fixed *CO*_2_ mass and the daily released *O*_2_ mass per unit leaf area of the plant can be calculated (g·m^2^·d^-1^). The formula for calculating the daily fixed *CO*_2_ mass is

$$W_{{CO}_{2}}=P∙44/1000$$ (2)

The formula for calculating the mass *O*_2_ released daily is

$$W_{O_{2}}=P∙32/1000$$ (3)

The formula for calculating the mass of *O*_2_ released daily is

Q=P∙LAI (4)

where *Q* is the daily net assimilation amount per unit area of the plant (mmol·m^2^·d^-1^), and *LAI* is the plant leaf area index.

Photosynthesis is a complex process, and photorespiration consumes a certain proportion of fixed *CO*_2_, which is generally believed to be 20–50% [41]. Therefore, the above formula for oxygen release and carbon fixation should also be multiplied by the photorespiration influence coefficient, and the median value for the calculation is 0.7 in this article. Appendix 1 in S1 File shows the family to which the tree belongs and its net assimilation data.

#### 2.3.2. Data collection of other indicators

Taking the year as the time scale, the C3–C15 indicator data from the evaluation system were collected in the following ways throughout 2022: (1) by conducting a comprehensive survey of the plants in Tianjin Qiaoyuan Park, counting the woody plant species, and assessing the growth conditions and landscape patterns in the park; (2) based on the existing relevant academic books [42,43] and an online query website (https://plants.usda.gov/home), the following data items were recorded for these varieties: habitat status, ecological habits, flowering period, fruiting period, branch form, flower shape, flower color, fruit shape and color, leaf shape and leaf color, crown shape, stress resistance, and distribution range. These data were subsequently entered into the statistical table in combination with written records, measurement data, and on-site photo shooting.

### 2.4. Construction of an evaluation index system

#### 2.4.1 Determination of the criterion layer

**Fig 4:**
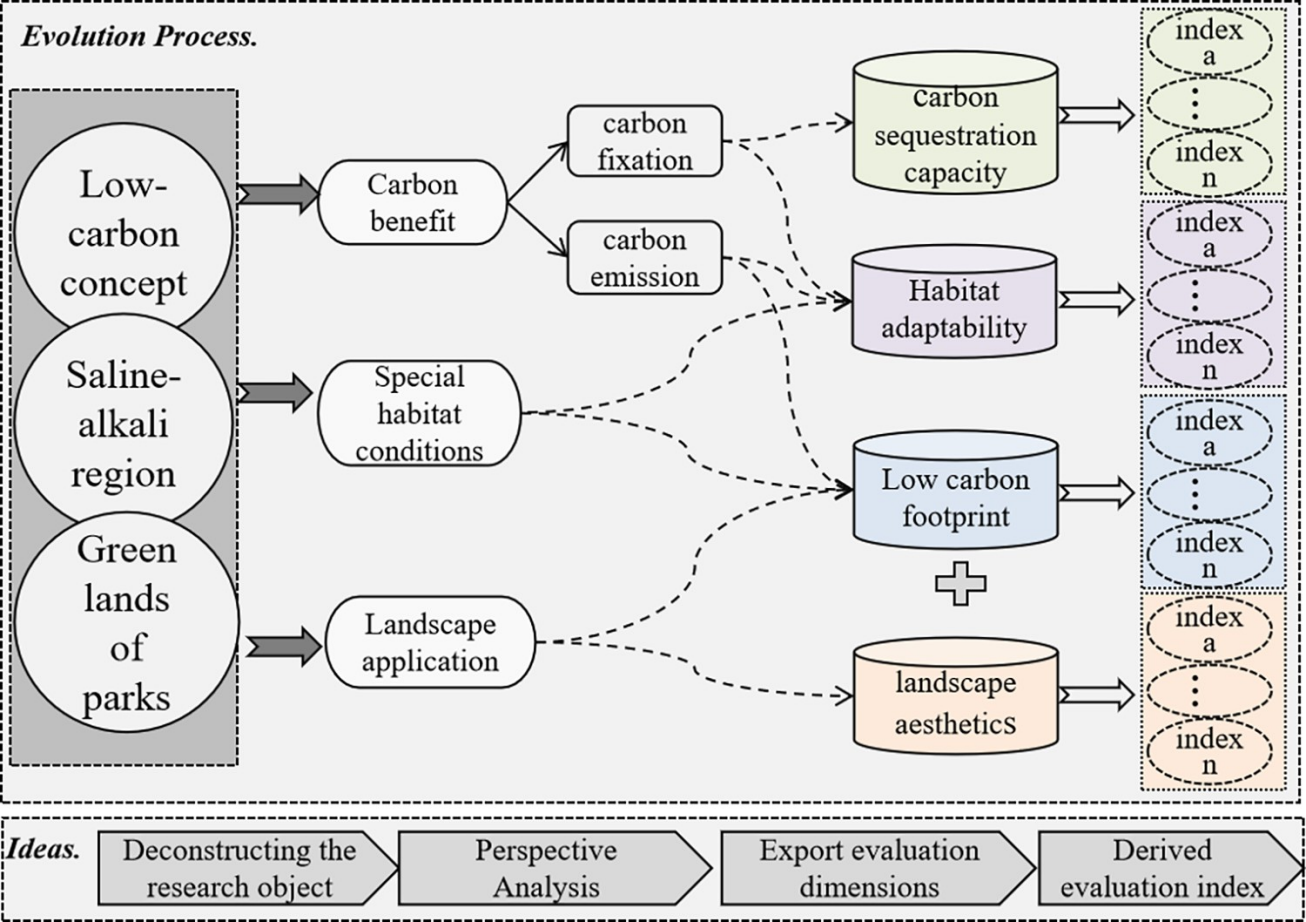
The evolution process of the concept map.

As shown in Fig 4, the research objectives of this article can be summarized into three topics, i.e., "Low-Carbon Concept", "Saline Land", and "Urban Park". In the construction of the criteria layer, the approach begins with the theory of sustainable development [44], which initially focuses on the "low-carbon concept." This emphasizes the critical role of urban parks in absorbing carbon dioxide and reducing the urban carbon footprint, leading to carbon sequestration capacity being proposed as the primary evaluation criterion.

Following this, based on the theory of species adaptability [45], the special habitat conditions of saline–alkali lands and their challenges to plant growth are considered, introducing habitat adaptability as the second evaluation criterion. Based on the principles of plant ecology, this criterion assesses the adaptability and survival of plant species in saline–alkali environments, and is directly linked to plant carbon sequestration efficiency and ecosystem stability. By analyzing the ecological characteristics of saline–alkali lands and their impact on plant growth, the importance of selecting plant species with strong adaptability for enhancing the ecological benefits of urban parks is highlighted.

Furthermore, based on the theory of the whole lifecycle, considering the potential carbon emissions from the construction and maintenance of urban parks, low carbon management and conservation is proposed as the third criterion. This study aimed to evaluate the carbon emissions at various stages of urban park design, construction, management, and maintenance by promoting the use of low-carbon technologies and methods to minimize the carbon footprint throughout the trees’ lifecycle.

Finally, based on landscape aesthetics theory [46] and recognizing that urban parks not only serve ecological and environmental functions but also possess significant landscape and aesthetic value [47], landscape aesthetics is incorporated into the evaluation system. This criterion emphasizes that urban parks should not only provide ecological services but also cater to people’s needs for a pleasant living environment, enhancing the aesthetic value and recreational function of urban parks through landscape design.

#### 2.4.2 Determination of indicator layer

**Fig 5:**
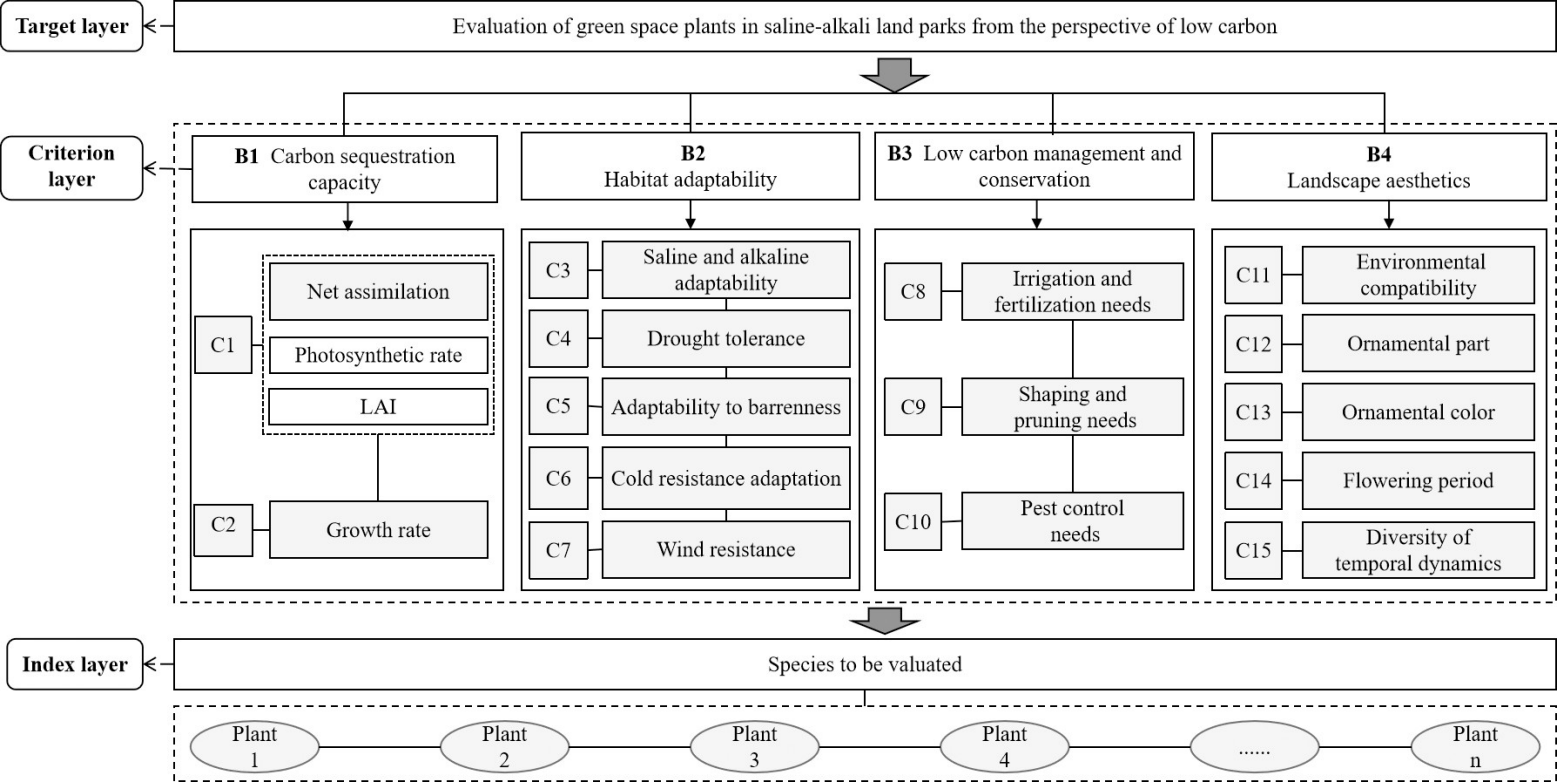
The hierarchical structure of the evaluation system.

In comparison to previous research, this article presents a comprehensive evaluation framework consisting of 15 assessment criteria (Fig 5). These criteria are organized into four categories: carbon sequestration capacity (B1), habitat Adaptability (B2), low-carbon management and conservation (B3), and landscape aesthetics (B4). We chose net assimilation (C1) and growth rate (C2) as indicators for evaluating the carbon sequestration capacity of plants according to previous studies [48,49]. Saline and alkaline adaptability (C3), drought tolerance (C4), adatability to barrenness (C5), cold resistance adaption (C6) and wind resistance (C7) were included in the evaluation system as subsidiary indicators of the saline habitat adaptation criteria layer according to the studies of Bao et al. [50] and Sun et al. [51]. We chose irrigation and fertilization needs (C8), shaping and pruning needs (C9) and pest control needs (C10) as subordinate indicators of planting and maintenance practices. Environmental compatibility (C11), ornamental part (C12), ornamental color (C13), flowering period and diversity of temporal dynamics (C14) were chosen to form the layer of aesthetic criteria of the landscape. The index of ornamental parts takes into account the simplicity and comprehensiveness of the index structure, flower viewing, fruit viewing, foliage viewing, and shape viewing [52].

This framework aims to better capture the specific requirements of park plants in adapting to saline and alkaline environments. Additionally, this study emphasizes the attributes of plants with high carbon sequestration levels and low carbon emissions. For each indicator, detailed descriptions and the basis of their sources are provided in Appendix 2 in S1 File.

Thirty senior engineers and technicians who had long been involved in the application of landscape plants were invited to convene a seminar to determine the scoring criteria for the indicators.

Table 1 displays the properties of the indicators, the criteria used for scoring, and the sources of the data. Compared to the previous evaluation index system construction [39,52–54], there was an observed increase in the proportion of quantitative indicators, reaching 73.33%. Therefore, the assessment of this study was characterized by a greater degree of objectivity and rigor.

**Table 1:** Comprehensive evaluation criteria for plants.

Target layer (A)	Criteria layer (B)	Indicator factor layer (C)	Attributes of indicators	The scoring criterion of the factor layer: excellent (80–100 points); medium (60–80 points; poor (<60 points)	Data Sources
A comprehensive evaluation of green space plants in saline–alkali areas from the perspective of low-carbon data sources (A1)	Carbon Sequestration Capacity (B1)	Net assimilation (C1)	Quantitative forward	Excellent: the measured value of net assimilation is above 700 mmol·m^2^·d^-1^; Medium: the measured value of net assimilation is 300–700 mmol·m^2^·d^-1^; 3) Inferior: the measured value of net assimilation is below 300 mmol·m^2^·d^-1^.	Instrument measurement.
		Growth rate (C2)	Quantitative forward	Excellent: the growth rate reaches more than 10 inches per year; Medium: the growth rate is between 2 inches and 10 inches per year; Inferior: the growth rate is less than 1 inch per year and less than 2 inches per year.	Online database (https://plants.usda.gov/home); Professional book "Manual of Woody Landscape Plants" [43].
	Habitat Adaptability (B2)	Saline and alkaline adaptability (C3)	Quantitative forward	Excellent: suitable soil pH ≥ 8.5; Medium: suitable soil 7.0 ≤pH<8.5; Inferior: suitable soil pH <7.0.	Online database (https://plants.usda.gov/home);Professional book "Manual of Woody Landscape Plants" [43], "China Halophyte Resources"[42].
		Drought tolerance(C4)	Qualitative positive	1) Excellent: most of them are strong positive tree species that can grow well under drought conditions; 2) Medium: moderate demand for water, able to survive under mild drought conditions; 3) Inferior: high water demand, mostly in moist soil such as at watersides.	Online database (https://plants.usda.gov/home);Professional book "Manual of Woody Landscape Plants" [43], "China Halophyte Resources" [42].
		Adaptability to barrenness (C5)	Qualitative positive	1) Excellent: It can maintain a good growth state in poor soil; 2) Medium: does not have strict requirements for soil; 3) Inferior: It can only thrive in fertile soil.	Online database (https://plants.usda.gov/home);Professional book "Manual of Woody Landscape Plants" [43],
		Cold resistance adaptation (C6)	Quantitative forward	1) Excellent: The temperature that it can safely survive in winter is below -20°C; 2) Medium: the safe winter temperature is between -10°C and -20°C; 3) Inferior: the safe winter temperature is above -10°C.	Online database (https://plants.usda.gov/home);Professional book "Manual of Woody Landscape Plants" [43]; "China Halophyte Resources"[42]; Site investigation.
		Wind resistance (C7)	Quantitative forward	1) Excellent: no branch breakage occurs; 2) Medium: only 1 point of branch breakage occurs; 3) Inferior: more than 2 points of branch breaking.	Professional book "Manual of Woody Landscape Plants" [43]; Literature Research [55,56]; Site investigation.
	Low-carbon management and conservation (B3)	Irrigation and fertilization needs (C8)	Quantitative negative	Excellent: plants have low water and nutrient requirements, and there is basically no need for irrigation or fertilization throughout the year; 2) Medium: plants need irrigation and fertilization for more than 1 month during the dry season or growth period; 3) Inferior: Plants need irrigation and fertilization for less than 1 month during the dry or growing season.	Online database (https://plants.usda.gov/home);Professional book "Manual of Woody Landscape Plants" [43].
		Shaping and pruning needs (C9)	Quantitative negative	1) Excellent: There is almost no need for pruning or pruning of dead branches throughout the year; 2) Medium: There are 1 or 2 seasons in a year that need pruning to maintain the plant’s neat appearance and healthy growth; 3) Inferior: pruning is required in the third quarter or the whole season to maintain a good growth state and a beautiful appearance.	Online database (https://plants.usda.gov/home);Professional book "Manual of Woody Landscape Plants" [43].
8		Pest Control Needs (C10)	Quantitative negative	Excellent: no pests under conventional management; Medium: fewer than 5 common pests and diseases; 3) Inferior: more than or equal to 5 common pests and diseases.	Online database (https://plants.usda.gov/home);Professional book "Manual of Woody Landscape Plants" [43];Literature Research [57,58];Site investigation.
	Landscape aesthetics (B4)	Environmental compatibility (C11)	Qualitative positive	1) Excellent: Generally, it is a local regional plant, and has a good sense of harmony with the environment of the saline–alkali land park; 2) Medium: native regional plants or naturalized plants, the harmony between the plants themselves and the saline–alkali land park environment is moderate; 3) Inferior: nonnative plants, the harmony between the plants themselves and the saline–alkali land park environment is poor.	Professional book "Tianjin Flora" [59], "China Halophyte Resources" [42];site investigation.
		Ornamental parts (C12)	Quantitative forward	1) Excellent: there are 2 or more ornamental parts with a noticeable ornamental period; 2) Medium: 1–2 ornamental parts with an apparent ornamental period; 3) Inferior: the appearance is the main focus, and there is no apparent viewing period.	Site investigation.
		Ornamental color (C13)	Qualitative positive	1) Excellent: the ornamental color is bright, eye-catching, and elicits a strong visual experience; 2) Medium: the ornamental color is brighter and allows for a stronger visual experience; 3) Inferior: the viewing color is not bright, and there is no strong visual experience.	Professional book "Manual of Woody Landscape Plants" [43];Site investigation.
		Flowering period (C14)	Quantitative forward	1) Excellent: the flowering period of the plant lasts for a long time, and there are flowers for more than 1 season or all year round; 2) Medium: the flowering period of the plant is moderate, from 1 month to 1 season; 3) Inferior: the plant’s flowering period is short, less than 1 month.	Online database (https://plants.usda.gov/home);Professional book "Manual of Woody Landscape Plants" [43];Site investigation.
		Diversity of temporal dynamics (C15)	Quantitative forward	Excellent: plants have significant landscape value throughout the year; Medium: plants have significant landscape value in 2 or 3 seasons; Inferior: the plants only have significant landscape value for 1 season.	Online database (https://plants.usda.gov/home);Professional book "Manual of Woody Landscape Plants" [43];Site investigation.

### 2.5. Determining the evaluation weight

#### 2.5.1. Determination of the weight of each evaluation level and the consistency test

In this study, the 1–9 reciprocal inverse scale method [60] was used to construct positive and negative judgment matrices, and the factors at each level were compared two by two and expressed numerically to form a matrix. Within the range of 1–9, the greater the number is, the greater the importance. The index used to measure the consistency of the judgment matrix is the CI:

$$CI=\left(\lambda_{max}-n\right)/\left(n-1\right)$$ (5)

Where n is the order of the matrix and λ_max_ is the largest characteristic root.

The index used to measure the consistency of the judgment matrix is the CR, which is the ratio of the CI to the random consistency index (RI) of the judgment matrix. The formula is as follows:

CR=CI/RI (6)

When CR<0.1, the judgment matrix has satisfactory consistency [61].

#### 2.5.2 Calculation of hierarchical total ranking weights

After calculating the weighted value of each evaluation index of index layer C relative to criterion layer B, it is combined with the weight of criterion layer B to obtain the total ranking weight of standard layer C relative to target layer A.

The formula for calculating the total ranking weight of the hierarchy is:

$$ci=\sum_{j=1}^{m}c_{ij}b_{j}(i=1,2,3\ldots n)$$ (7)

b1, b2, b3…bm are the single-ranking weights of the previous level B; cij…cnj are the single-ranking weights of Bj in the current level C; and *c*1,*c*2,*c*3…*cn* are the total ranking weights of each factor in the C level.

### 2.6. Clustering and ranking

The existing clustering methods fall into five main types: division-based, grid-based, density-based, hierarchical, and model-based clustering algorithms [62]. Among them, hierarchical clustering methods can generate a hierarchical clustering structure without predefining the number of clusters, provide intuitive dendrogram visualization, or exhibit better processing capabilities for multidimensional data and outliers [63]. Complete linkage clustering (CLC) is a systematic clustering method that can provide clear intercluster boundaries, is robust to outliers, and handles nonspherical and multidimensional data characteristics well [64]; moreover, this method is suitable for use as a clustering method in this study.

According to Liu’s study of the intermediate upper quartile and lower quartile as grade split points [65], the composite plant scores were categorized into three classes. The specific calculation formula is as follows:

$$Q_{1}=\left(n+1\right)/4$$ (8)

$$Q_{3}=3*(n+1)/4$$ (9)

*Q*_1_ is the first quartile, *Q*_3_ is the third quartile, and *n* is the total number of data points.

### 2.7. Analysis and plotting software

This study utilized R language (version 3.6.3) for all data analysis and visualization tasks. Data processing and analysis included the AHP and cluster analysis, implemented through the ‘ahp’ and ‘stats’ packages, respectively. Visualizations were produced using the ‘ggplot2’ package, with heatmaps created by the ‘geom_tile()’ function to display inter-variable correlations; boxplots were generated using the ‘geom_boxplot()’ function to compare data distributions and outliers across groups. Furthermore, all analyses were conducted in an RStudio environment on a 64-bit Windows 10 operating system, ensuring the reproducibility of the processing workflow.

## 3. Results

### 3.1. Evaluation index weights for the consistency test results

Five judgment matrices were constructed: A—(B1~B4), B1—(C1~C2), B2—(C3~C7), B3—(C8~C10), and B4—(C11-C15). Through analysis with MATLAB software, the results showed that the CR values of all five judgment matrixes were less than 0.1, indicating that they all possessed consistency (Table 2).

**Table 2:** Results of the consistency test.

Model hierarchy	Judgement matrix			
A-B	A	w^1^	λmax^2^	CR^3^
	B1	0.277	4.031	0.016
	B2	0.466		
	B3	0.161		
	B4	0.096		
B1-C	B1	w	λmax	CR
	C1	0.750	-	0.000
	C2	0.250		
B2-C	B2	w	λ_max_	CR
	C3	0.408	-	0.000
	C4	0.272		
	C5	0.136		
	C6	0.102		
	C7	0.082		
B3-C	B3	w	λmax	CR
	C8	0.493	5.000	0.000
	C9	0.296		
	C10	0.211		
B4-C	B4	w	λmax	CR
	C11	0.132	5.258	0.000
	C12	0.066		
	C13	0.264		
	C14	0.262		
	C15	0.276		

^1^ w represents the weight value. ^2^ λmax represents the largest characteristic root. ^3^ CR represents the consistency ratio of the judgment matrix.

From Table 2, it is evident that in the criteria layer, the weight values are B2 > B1 > B3 > B4, indicating that the habitat adaptability of the trees is of utmost importance, and the carbon sequestration capacity of trees also needs to be prioritized.The combined weights of the evaluation indicators in the indicator layer were ranked as follows: cold resistance adaptation (0.048) = shaping and pruning needs (0.048) > wind resistance (0.038) > pest control needs (0.034) > diversity of temporal dynamics (0.026) > ornamental color (0.025) = flowering period (0.025) > environmental compatibility (0.013) > ornamental parts (0.006).

### 3.2. Analysis of clustering results of individual index scores

**Fig 6:**
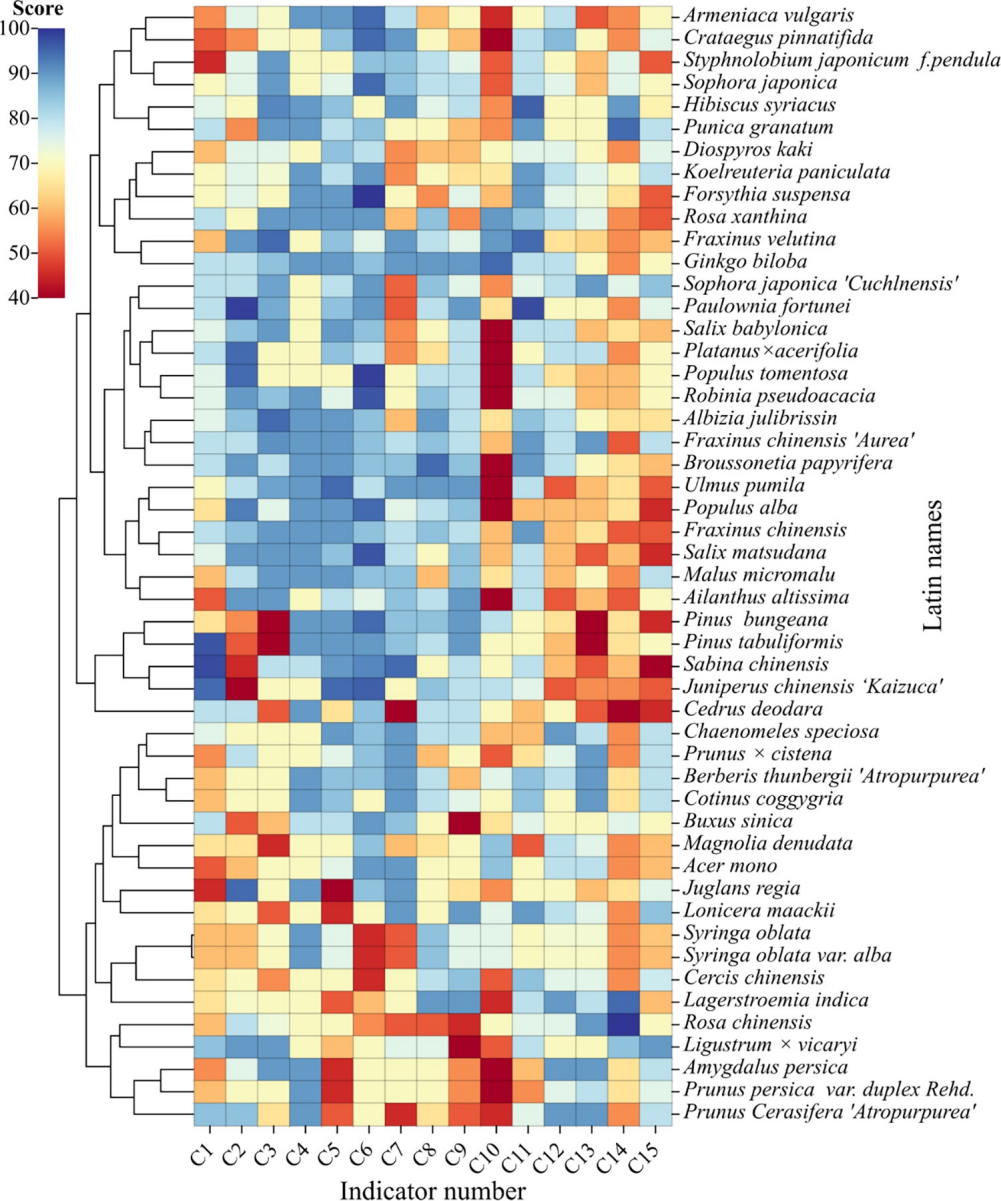
Heatmap and cluster analysis of evaluation scores or individual indicators of the 50 researched plants.

Fig 6 shows the evaluation scores or individual indicators of the 50 researched plants. According to the clustering results, the 50 woody plant species investigated in this study were divided into five categories: *Pinus bungeana*, *Pinus tabuliformis*, *Juniperus chinensis* ’Kaizuca’, and *Sabina chinensis* are in the first category. These species generally exhibit excellent net assimilation ability and high tolerance to drought, barrenness, and wind but exhibit poor performance in terms of ornamental parts, ornamental colors, flowering period length, and temporal diversity.

*Cedrus deodara*, *Fraxinus velutina*, *Ginkgo biloba*, and *Rosa xanthina* are in the second category; these species perform better in terms of net assimilation and growth rate and better in the subordinate metrics of habitat adaptability and habitat adaptability and moderate performance in the subordinate indices of landscape aesthetics.

*Sophora japonica* ’Cuchlnensis’, *Paulownia fortune*i, *Salix babylonica*, *Platanus* × *Acerifolia*, *Populus tomentosa*, *Robinia pseudoacacia*, *Albizia julibrissin*, *Fraxinus chinensis* ’Aurea’, *Broussonetia papyrifera*, *Ulmus pumila*, *Populus alba*, *Fraxinus chinensis*, *Salix matsudana*, *Malus micromalu*, *Ailanthus altissima*, *Armeniaca vulgaris*, *Crataegus pinnatifida*, *Styphnolobium japonicum* f. *pendula*, *Sophora japonica*, *Hibiscus syriacus*, and *Punica granatum* are in the third category; these species perform very poorly in pest and disease control needs and poorly in terms of the subordinate indicators of landscape aesthetics, but all perform better in terms of the subordinate indicators of habitat suitability, namely, saline and alkaline adaptability, drought tolerance, adaptability to barrenness and cold tolerance. All of them also performed better in terms of growth rate.

In the fourth category, we included *Diospyros kaki*, *Koelreuteria paniculata*, *Forsythia suspensa*, *Buxus sinica*, *Chaenomeles speciosa*, *Prunus* × *cistena*, *Berberis thunbergii* var. *atropurpurea Chenault*, *Cotinus coggygria*, *Magnolia denudata*, *Acer mono*, *Juglans regia*, *Lonicera maackii*, *Syringa oblata*, *Syringa oblata* var. *alba*, *Cercis chinensi*s, and *Lagerstroemia indica*. These species perform well in terms of drought tolerance, cold tolerance, and environmental compatibility, with average performance for the remaining indicators. *Rosa chinensis*, *Ligustrum* × *vicaryi*, *Prunus persica* ‘Alropurpurea’, *Prunus persica*, and *Prunus cerasifera ’Atropurpurea’* were in the fifth category. These tree species showed better landscape aesthetic value, especially for the three indicators of ornamental parts, ornamental colors, and temporal dynamic diversity, and they also exhibited better salinity and alkalinity adaptability and drought tolerance. However, they scored very low in terms of the two indicators of shaping and pruning needs and pest and disease control needs.

### 3.3. Comprehensive evaluation score grading results

After the final weight values were combined to calculate the composite score, the results were divided into three grades according to the 50 woody plants. Table 3 shows that Grade I ≥ 79.59, 79.59 > Grade II ≥ 69.27, and Grade III < 69.27.

**Table 3:** Comprehensive evaluation scores and classification.

Rangking	Latin name	Type of tree	Overall ratings	Grades
1	*Fraxinus chinensis ’Aurea’*	Deciduous tree	82.805	Ⅰ
2	*Broussonetia papyrifera*	Deciduous tree	82.611	Ⅰ
3	*Fraxinus chinensis*	Deciduous tree	81.78	Ⅰ
4	*Albizia julibrissin*	Deciduous tree	81.75	Ⅰ
5	*Ginkgo biloba*	Deciduous tree	81.275	Ⅰ
6	*Rosa xanthina*	Deciduous shrub	81.165	Ⅰ
7	*Ulmus pumila*	Deciduous tree	80.565	Ⅰ
8	*Hibiscus syriacus*	Deciduous shrub	80.546	Ⅰ
9	*Juniperus chinensis ‘Kaizuca’*	Evergreen tree	80.013	Ⅰ
10	*Sabina chinensis*	Evergreen tree	79.83	Ⅰ
11	*Salix matsudana*	Deciduous tree	79.671	Ⅰ
12	*Robinia pseudoacacia*	Deciduous tree	79.591	Ⅰ
13	*Paulownia fortunei*	Deciduous tree	78.964	Ⅱ
14	*Sophora japonica ’Cuchlnensis’*	Deciduous tree	78.888	Ⅱ
15	*Punica granatum*	Deciduous tree	78.475	Ⅱ
16	*Sophora japonica*	Deciduous tree	77.82	Ⅱ
17	*Salix babylonica*	Deciduous tree	76.782	Ⅱ
18	*Malus micromalu*	Deciduous tree	76.455	Ⅱ
19	*Fraxinus velutina*	Deciduous tree	76.43	Ⅱ
20	*Populus alba*	Deciduous tree	76.03	Ⅱ
21	*Ligustrum × vicaryi*	Evergreen shrub	75.71	Ⅱ
22	*Berberis thunbergii ’Atropurpurea’*	Deciduous shrub	75.565	Ⅱ
23	*Pinus tabuliformis*	Evergreen tree	74.956	Ⅱ
24	*Koelreuteria paniculata*	Deciduous tree	74.889	Ⅱ
25	*Chaenomeles speciosa*	Deciduous shrub	74.42	Ⅱ
26	*Forsythia suspensa*	Deciduous shrub	73.26	Ⅱ
27	*Platanus×acerifolia*	Deciduous tree	73.245	Ⅱ
28	*Cotinus coggygria*	Deciduous tree	72.92	Ⅱ
29	*Ailanthus altissima*	Deciduous tree	72.035	Ⅱ
30	*Populus tomentosa*	Deciduous tree	71.522	Ⅱ
31	*Amygdalus persica*	Deciduous tree	71.495	Ⅱ
32	*Buxus sinica*	Evergreen shrub	71.43	Ⅱ
33	*Styphnolobium japonicum f*.*pendula*	Deciduous tree	71.38	Ⅱ
34	*Syringa oblata*	Deciduous shrub	71.363	Ⅱ
35	*Prunus Cerasifera ’Atropurpurea’*	Deciduous tree	70.795	Ⅱ
36	*Armeniaca vulgaris*	Deciduous tree	70.615	Ⅱ
37	*Pinus bungeana*	Evergreen tree	70.035	Ⅱ
38	*Diospyros kaki*	Deciduous tree	69.27	Ⅱ
39	*Syringa oblata var*. *alba*	Deciduous shrub	69.046	Ⅲ
40	*Acer mono*	Deciduous tree	68.68	Ⅲ
41	*Lagerstroemia indica*	Deciduous shrub	68.67	Ⅲ
42	*Prunus × cistena*	Deciduous tree	67.83	Ⅲ
43	*Crataegus pinnatifida*	Deciduous tree	67.691	Ⅲ
44	*Prunus persica var*. *duplex Rehd*.	Deciduous tree	67.51	Ⅲ
45	*Cedrus deodara*	Evergreen tree	67.19	Ⅲ
46	*Juglans regia*	Deciduous tree	66.21	Ⅲ
47	*Lonicera maackii*	Deciduous tree	65.934	Ⅲ
48	*Cercis chinensis*	Deciduous tree	65.853	Ⅲ
49	*Magnolia denudata*	Deciduous tree	65.61	Ⅲ
50	*Rosa chinensis*	Deciduous shrub	65.505	Ⅲ

The total number of plants with a combined Grade I score was 12. There are two kinds of evergreen trees, Sabina chinensis and Juniperus chinensis ’Kaizuca’; 8 types of deciduous trees, Fraxinus chinensis ’Aurea’, Broussonetia papyrifera, Fraxinus chinensis, Albizia julibrissin, Ginkgo biloba, Ulmus pumila, *Salix matsudana*, and Robinia pseudoacacia; and two kinds of deciduous shrubs, Hibiscus syriacu and Rosa xanthina. Grade I plants are mostly characterized by high comprehensive evaluation and are characterized by strong carbon sequestration and oxygen release capacities, good habitat adaptability, easy management and maintenance, and high landscape application value; they can also be used as the preferred tree species for the low-carbon construction of parks and green spaces in the Bohai Sea region.

A total of 26 plant species had a comprehensive Grade II scores. There are 2 kinds of evergreen trees, namely, *Pinus tabuliformis* and *Pinus bungeana*; 18 kinds of deciduous trees, including Paulownia fortunei, Sophora japonica ’Cuchlnensis’, Punica granatum, Sophora japonica, Salix babylonica, Malus micromalu, Fraxinus velutina, Populus alba, Koelreuteria paniculata, Platanus×acerifolia, Cotinus coggygria, Populus tomentosa, *Prunus persica* ’Alropurpurea’, Styphnolobium japonicum f. pendula, Prunus Cerasifera ’Atropurpurea’, Armeniaca vulgaris, and Diospyros kaki; 2 kinds of evergreen shrubs, namely, Ligustrum × vicaryi and Buxus sinica; and 4 kinds of deciduous shrubs, including Berberis thunbergii var. atropurpurea Chenault, Chaenomeles speciosa, Forsythia suspensa, and Syringa oblata. The Grade II plants are rated relatively high in terms of their aesthetic value, adaptability, management and maintenance, and carbon sequestration capacity and have good application value. It is recommended that these materials be developed and popularized for use in promoting the low-carbon development of green construction in the Bohai Bay region and enhancing its landscape aesthetics.

A total of 12 plants had a combined Grade III score. Among these, one species of evergreen tree was found, namely, Cedrus deodara; eight species of deciduous trees, namely, Acer mono, Prunus × cistena, Crataegus pinnatifida, *Prunus persica*, Juglans regia, Lonicera maackii, Cercis chinensis, and Magnolia denudata; and three species of deciduous shrubs, namely, Syringa oblata var. alba, Lagerstroemia indica, and Rosa chinensis. The Grade III plants have a low comprehensive score and can be selectively used in constructing the Bohai Bay area. For example, Magnolia denudata, Cercis chinensis, Crataegus pinnatifida, and Prunus Cerasifera ’Atropurpurea’ are better at the ornamental level. However, these plants perform poorly in terms of low-carbon pipe maintenance and are generally resistant to salinity and alkali, so they should be applied appropriately at important ornamental nodes.

**Fig 7:**
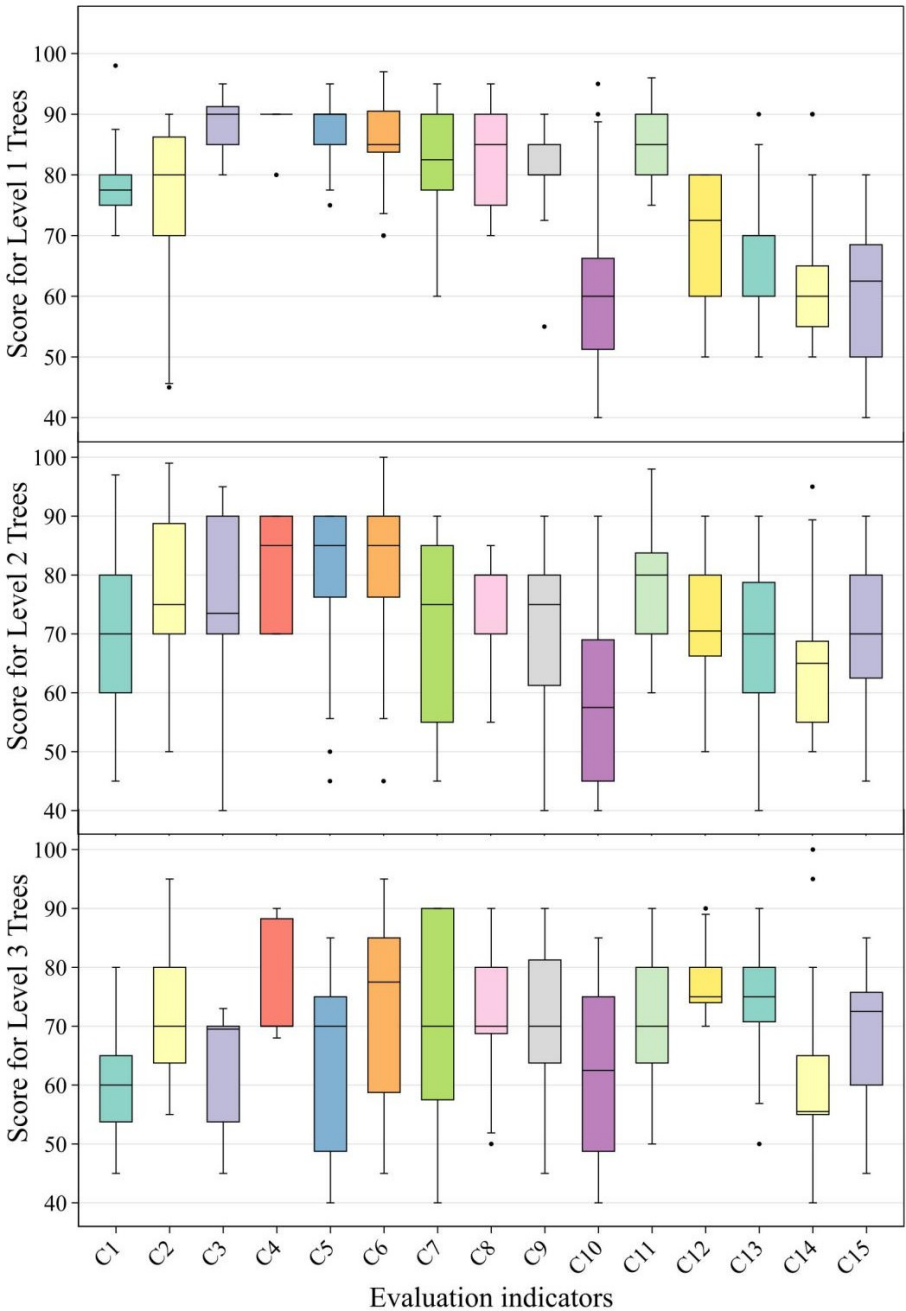
Scores for indicators across different grades of tree species.

A box plot of the scores at the indicator level for plants of various grades generated is shown in Fig 7. There are a total of 12 species of Grade I trees, including 2 evergreen trees, 8 deciduous trees, and 2 deciduous shrubs. The Grade I trees performed better in terms of carbon sequestration and oxygen release capacity as well as habitat adaptability at the criteria layer, which was particularly evident in indicators such as growth rate, salt-alkali resistance, and drought tolerance. They also generally require less pruning, making them a priority choice for the low-carbon construction of parks and green spaces in the Tianjin area.

There were total of 26 Grade II trees, including 2 evergreen trees, 18 deciduous trees, 2 evergreen shrubs, and 4 deciduous shrubs. The Grade II trees exhibited excellent performance at the landscape aesthetic level and exhibited relatively good adaptability, management and maintenance, and carbon sequestration capabilities. It is recommended that their application be developed and promoted, especially at green nodes with high landscape requirements.

The Grade III trees consisted of 12 species, including 1 evergreen tree, 8 deciduous trees, and 3 deciduous shrubs. Overall, Grade III trees had lower scores, particularly in terms of carbon sequestration benefits and carbon emission levels. However, some species exhibit long flowering periods and more appealing ornamental colors, and can be selectively applied to important ornamental nodes. For instance, *Cercis chinensis* has a long blooming period and bright flower colors, making it suitable for embellishment at key landscape nodes such as road intersections.

## 4. Discussion

### 4.1. The scientific and rationality of the evaluation criteria system

This study utilized AHP method to construct an evaluation system that includes four criteria: carbon sequestration capacity, habitat suitability, low carbon management and conservation, and landscape aesthetics. In the fields of environmental management and urban greening, AHP is suitable for assessing the relative importance of multiple factors and criteria [66]. Wei et al. applied the Analytic Hierarchy Process (AHP) to develop an evaluation system for plants well-adapted to coastal saline–alkali soils, incorporating criteria such as habitat adaptability, ecological resilience, and visual appeal [67]. They conducted a selection of suitable plant species for the Yingwuzhou Wetland Park in Jinshan New City, Shanghai. Our study emphasized additional metrics associated with the plants’ low-carbon properties.

In this study, the five indicators of drought tolerance, cold tolerance, salinity tolerance, barrenness tolerance, and wind tolerance were included in the evaluation system as subsidiary indicators of the saline habitat adaptation criteria layer, which is similar to the selection of salinity tolerance, wind tolerance, and drought tolerance by Sun Lihui et al. as the factors influencing the selection of plants for coastal saline–alkaline land [51]. Li et al. argued that net assimilation rate effectively demonstrates the carbon sequestration capacity of individual tree species [68]. Zaid et al. believed that the growth rate of plants indirectly affects their carbon sequestration ability [48]. This study selects these two factors as indicators for evaluating the carbon sequestration capacity of plants. Previous studies often used maintenance costs as a criterion within the indicator layer, for example, Tan et al. included maintenance management as a subordinate indicator under the planting and care criteria layer [69]. This study elevated maintenance management to a criterion layer, with subordinate indicators including irrigation and fertilizer requirements, shaping and pruning, and pest and disease control. The aim was to provide a more detailed assessment of the carbon footprint generated during the plant maintenance process.

Compared with the results of the criterion layer weight allocation in existing studies related to the evaluation of greenfield plants [54,69,70] a higher weight was assigned to the habitat adaptability criterion layer. This prioritization is due to the fact that, in saline-alkali environments, tolerance to such conditions is a fundamental prerequisite for the survival of tree species. Furthermore, this emphasis aligns with the findings of Bao et al., who argued that plants well-adapted to their native ecosystems can demonstrate enhanced carbon sequestration capabilities, thereby reducing both conservation and management needs as well as carbon emissions [50]. Although the weight attributed to landscape aesthetics has been reduced, its significant role in boosting the social value and public acceptance of parks cannot be disregarded and must be factored into practical geening applications.

As mentioned above, the evaluation system constructed in this study differed from the previous indicators and weights used for saline-alkali tolerant plants in urban green spaces, the evaluation system placed more emphasis on environmental adaptability and the complexity of planting and maintenance under the special environmental conditions of saline–alkaline land, while the ornamental and application values were secondarily important, which was in line with the principles of carbon sequestration by plants as well as greening requirements in the ecological and environmental context of saline–alkaline land.

### 4.2. Optimization of plant selection and application strategies

The research results indicate that the carbon sequestration capacity of plants in Qiaoyuan is slightly lower than reported in studies by Jin et al. [71] and Chen et al. [72]. This may be attributed to the poor soil quality and dry climate conditions in the area, which could lead to a decline in plant health and consequently reduce their carbon sequestration efficiency. The study also found that tree species with strong salt-alkaline resistance often possess strong carbon sequestration capabilities and are commonly native species [59]. This may be related to the fact that native plants are highly tolerant and typically show good ecological adaptability to the local environment [73]. Native trees have lower collection and handling costs and emit less carbon, while exotic plants have higher transportation costs and emit more carbon [74]. Therefore, priority should be given to the use of native plants, which not only reduces management costs but also protects native plant resources. Native plants, such as *Fraxinus velutina*, *Ginkgo biloba*, *Sophora japonica*, and *Punica granatum*, had high overall scores and a more even distribution of index scores.

Morkovina’s findings confirm that a combination of fast-growing and slow-growing plants can significantly increase the carbon sequestration capacity of plant communities [75]. The optional fast-growing species included *Ailanthus altissima*, *Sophora japonica*, *Sophora japonica* ’Cuchlnensis’, *and Salix matsudana*, and the optional slow-growing species included *Pinus tabuliformis*, *Pinus bungeana*, *Sidewinder*, *Sabina chinensis*, *and Ginkgo biloba*. The selection of a plant community composed of highly carbon-sequestering tree species was performed in this study: *Sabina chinensis*+ *Fraxinus chinensis* ’Aurea’+ *Broussonetia papyrifera*+ *Prunus Cerasifera* ’Atropurpurea’+ *Lagerstroemia indica*+ *Buxus sinica*. The combination of evergreen plants with colorful and deciduous plants and the fast-growing species *Sophora japonica* and *Salix babylonica* create a landscape with high carbon sequestration levels while forming a park community that can be viewed throughout all seasons.

While grasping the law of plant carbon sequestration, saline and alkal tolerant plants with high ornamental value and good greening effects should also be used to construct landscaped green areas [76]. For example, *Juniperus chinensis ’Kaizuca’+ Populus tomentosa+ Sophora japonica* ’Cuchlnensis’*+ Pinus bungeana+ Malus micromalu+ Koelreuteria paniculata+ Forsy hia suspensa+ Syringa oblata* can form a seasonal landscape with flowers in spring (*Forsythia suspensa*, *Syringa oblata*), foliage in summer (*Sophora japonica* ‘Cuchlnensis’, *Juniperus chinensis* ‘Kaizuca’), fruits in autumn (*Malus micromalu*, *Koelreuteria paniculata*), and branches in winter (P*opulus tomentosa*, *Pinus bungeana*), while at the same time compensating for the lack of carbon efficiency among the plants.

### 4.3. Limitations and future prospects

Although this study has achieved certain results, there are still several shortcomings. First, there are more types of saline and alkaline areas with wider distribution ranges. The evaluation system constructed in this study, with only one saline park as an example, is not applicable to the evaluation of park green space plants in all saline areas. Second, the carbon efficiency data of the trees measured from June to September represent only the period when the plants have greater carbon sequestration capacity and are more obviously stressed by saline–alkali conditions. However, data from other seasons should not be overlooked either。

Against the backdrop of global climate change and ecological restoration, the importance of greening and low-carbon management of saline–alkali lands is increasingly highlighted. Future research should transcend the limitations of single regions or countries to explore the adaptability and carbon sequestration potential of saline–alkali land plants under different geographical and climatic conditions. This includes comparative analysis of plant species and management strategies that have been successfully applied in saline–alkali environments worldwide, as well as their contributions to enhancing ecosystem services and carbon sequestration. Further research should also include a whole year data monitoring to gain a deeper understanding of the carbon capture capabilities of saline–alkali land plants and their performance throughout their life cycle. Moreover, interdisciplinary collaboration and technological innovation are crucial for addressing the challenges of greening and low-carbon management in saline–alkali lands. Future studies should promote cooperation among experts from different fields, utilizing big data and artificial intelligence technologies to optimize the selection and management of saline–alkali land plants, providing scientific support for sustainable management.

## 5. Conclusion

In this study, the hierarchical analysis method was used to determine the primary role of carbon in plants via a plant evaluation system. A comprehensive system for evaluating plants in saline–alkali parks was constructed considering four aspects, namely, carbon sequestration, carbon emission, adaptive capacity, and landscape aesthetics, to comprehensively evaluate the 50 species of woody plants in Tianjin Qiaoyuan Park. According to the comprehensive evaluation scores in the four aspects, the 50 plant species were divided into three grades. Grade I including 13 plants, such as *Rosa xanthina and Robinia pseudoacacia*. exhibit the great carbon sequestration capabilities and salt-alkaline resistance, along with relatively lower carbon emissions. These characteristics make them suitable as priority plant species.Grade II plants encompass 24 species, which exhibited good carbon sequestration and aesthetic value, with moderate carbon emissions. They can serve as alternative options to enrich urban plant diversity.Grade III comprising 13 species, tends to have higher carbon emissions due to regular maintenance and exhibit average habitat adaptability. Thus, tree species in Grade I and II are recommended in the implementation of low-carbon greening projects in the Bohai Bay region, while Grade III tree species should be judiciously utilized. Our methods and findings helps to scientifically assess the comprehensive quality of green space plants, provides a basis and reference for subsequent greening work, and promotes the sustainable development of urban construction in saline areas.

## Supporting information

S1 File (DOCX)

S2 File (ZIP)
