# Supplementary material for: Comprehensive evaluation and application of woody plants in the green spaces of parks in saline–Alkaline areas from a low-carbon perspective: A case study of Tianjin Qiaoyuan Park
Source: PLoS One. 2024 May 10;19(5):e0303341. doi: 10.1371/journal.pone.0303341 (PMC11086879; doi:10.1371/journal.pone.0303341)
Supplement: S1 File — (DOCX) [file pone.0303341.s001.docx]

**Appendix 1.**

Table 1 shows the data for each tree family and genus and the net assimilation data.

**Table 1**. Data related to plant carbon sequestration.

| **Latin name** | **Net assimilation（mmolm^-2^d^-1^）** | **Ranking** | **Latin name** | **Net assimilation (mmolm^-2^d^-1^)** | **Ranking** |
| --- | --- | --- | --- | --- | --- |
| *Sabina chinensis* | 1651.06 | 1 | *Koelreuteria paniculata* | 475.75 | 26 |
| *Pinus tabuliformis* | 1597.19 | 2 | *Ulmus pumila* | 470.03 | 27 |
| *Juniperus chinensis ‘Kaizuca'* | 1506.37 | 3 | *Cercis chinensis* | 465.60 | 28 |
| *Ligustrum × vicaryi* | 1121.71 | 4 | *Pinus bungeana* | 453.07 | 29 |
| *Buxus sinica* | 939.58 | 5 | *Lagerstroemia indica* | 443.96 | 30 |
| *Prunus Cerasifera 'Atropurpurea'* | 903.92 | 6 | *Populus alba* | 439.46 | 31 |
| *Punica granatum* | 890.77 | 7 | *Berberis thunbergii 'Atropurpurea'* | 437.73 | 32 |
| *Ginkgo biloba* | 805.7 | 8 | *Magnolia denudata* | 427.26 | 33 |
| *Paulownia fortunei* | 804.3 | 9 | *Rosa chinensis* | 401.76 | 34 |
| *Fraxinus chinensis* | 787.52 | 10 | *Syringa oblata* | 387.43 | 35 |
| *Fraxinus chinensis 'Aurea'* | 784.58 | 11 | *Lonicera maackii* | 382.80 | 36 |
| *Sophora japonica 'Cuchlnensis'* | 784.45 | 12 | *Syringa oblata var. alba* | 369.70 | 37 |
| *Rosa xanthina* | 758.20 | 13 | *Prunus persica var. duplex Rehd.* | 334.19 | 38 |
| *Broussonetia papyrifera* | 756.40 | 14 | *Fraxinus velutina* | 317.88 | 39 |
| *Platanus×acerifolia* | 743.95 | 15 | *Cotinus coggygria* | 295.89 | 40 |
| *Cedrus deodara* | 731.46 | 16 | *Malus micromalu* | 281.29 | 41 |
| *Albizia julibrissin* | 662.75 | 17 | *Armeniaca vulgaris* | 269.54 | 42 |
| *Hibiscus syriacus* | 622.74 | 18 | *Prunus × cistena* | 256.90 | 43 |
| *Chaenomeles speciosa* | 604.5 | 19 | *Amygdalus persica* | 254.87 | 44 |
| *Sophora japonica* | 584.07 | 20 | *Acer mono* | 232.53 | 45 |
| *Salix babylonica* | 549.03 | 21 | *Crataegus pinnatifida* | 203.64 | 46 |
| *Robinia pseudoacacia* | 545.34 | 22 | *Ailanthus altissima* | 182.24 | 47 |
| *Salix matsudana* | 523.60 | 23 | *Diospyros kaki* | 161.23 | 48 |
| *Populus tomentosa* | 501.33 | 24 | *Styphnolobium japonicum f.pendula* | 92.18 | 49 |
| *Forsythia suspensa* | 494.36 | 25 | *Juglans regia* | 91.08 | 50 |

**Appendix 2 .**

The text description and source basis of each indicator are shown in Table 2.

**Table 2.** The basis for the sources of indicators.

|  | Indicator name | Text description | Source basis |
| --- | --- | --- | --- |
| Net assimilation（C1） | Photosynthetic rate | The higher the photosynthetic rate, the more carbon the plant fixes per unit of time. | Fares, S., Paoletti, E., Calfapietra, C., et al. (2017) ^[1]^ |
|  | LAI | The greater the leaf area index, the more sunlight intercepted by plants, and the greater the intensity of photosynthesis. | Xue, X., Jinchi, Z., Yongtao, S., Jiayao, Z. and Yingxiang, W (2016) ^[2]^ |
| Growth rate (C2) | | The growth rate of plants is one of the factors affecting the total carbon sequestration of plants; fast-growing plants require more carbon dioxide to support their growth. Therefore, they generally have a higher carbon sequestration capacity. | Zaid, S. M., Perisamy, E., Hussein, H., et al. (2018) ^[3]^  Zhao, M., Kong, Z., Escobedo, F. J., et al. (2010) ^[4]^ |
| Salt–alkali adaptability (C3) | | The soil in saline–alkali land usually contains high-salinity and alkaline substances, and only plants with a certain degree of saline–alkali tolerance can survive and reproduce in this environment. | Song, X., Su, Y., Zheng, J., et al. (2022) ^[5]^ |
| Drought tolerance adaptability (C4) | | Drought has been identified as a major limiting factor for plant growth in desert regions. | Chang, Y., Fan, Y., Li, Z., et al. (2022) ^[6]^ |
| Adaptability to barrenness  s (C5) | | The soil in saline–alkali land is often nutrient-poor. In addition to its high salt content, it may lack the nutrient elements necessary for plant growth; plant photosynthesis is disturbed by sudden low-nutrient effects. | Linhui, D. (2020) ^[7]^  Korrensalo, A., Alekseychik, P., Hájek, T., et al. (2017) ^[8]^ |
| Cold resistance adaptation (C6) | | The temperature in Tianjin is low in winter, so it is necessary to take cold-proof measures for trees. | Yan, Z. (2010) ^[9]^ |
| Wind resistance (C7) | | There are many winds in Tianjin area, so it is necessary to take necessary wind protection measures for trees. | Sun, L., Zhang, J. H. (2013) ^[10]^ |
| Irrigation and fertilization needs  (C8) | | Production and transportation during fertigation require energy consumption and create a carbon footprint. | Haehle, R. G., Brookwell, J. N. (2004) ^[11]^ |
| Shaping and  pruning needs  (C9) | | The frequent pruning of plants will cause a large amount of pollutant emissions. | Yuan, H. F., Zhang, Y., Xu, W. B., et al. (2023) ^[12]^ |
| Pest control  needs (C10) | | Controlling plant pests and diseases may lead to a certain amount of carbon emissions. | Bao, Z. Y., Ma, J. T. (2011) ^[13]^ |
| Environmental compatibility  (C11) | | The environmental harmony of plants can reflect the relationship between plants and saline habitats. | Liu, R. X., Peng, Y. Y. (2017) ^[14]^ |
| Ornamental  parts (C12) | | The shape of plants, including the tree shape, leaf shape, flower shape, etc., are all critical factors affecting their ornamental value. | Bell, S. (2019) ^[15]^ |
| Ornamental  color (C13) | | Plants convey visual beauty through color. | Daniel, T.C. (2001) ^[16]^ |
| Flowering  period (C14) | | The flowering period of plants affects the ornamental value of garden plants; selecting plants with long flowering periods, bright flowers, and bright fruits can increase the color change and visual impact. | Callicott, J B. (1983) ^[17]^ |
| Diversity of  temporal dynamics  (C15) | | Plants also exhibit unique color effects due to seasonal changes and growth from juvenile to adult. | Wang, Z., Li, M. Y. (2017) ^[18]^ |

References

[1] PEARLMUTTER D, CALFAPIETRA C, SAMSON R, et al. The Urban Forest [J]. Cultvating Green Infrastructure for People, 2017,

[2] XUE X, JINCHI Z, YONGTAO S, et al. Study of carbon seqestration ＆ oxygen release and cooling ＆ humidifying effect of main greening tree species in Shanghai [J]. Journal of Nanjing Forestry University ( Natural Sciences Edition）, 2016, 59(03): 81.

[3] ZAID S M, PERISAMY E, HUSSEIN H, et al. Vertical Greenery System in urban tropical climate and its carbon sequestration potential: A review [J]. Ecological Indicators, 2018, 91(57-70.

[4] ZHAO M, KONG Z-H, ESCOBEDO F J, et al. Impacts of urban forests on offsetting carbon emissions from industrial energy use in Hangzhou, China [J]. Journal of environmental management, 2010, 91(4): 807-13.

[5] SONG X, SU Y, ZHENG J, et al. Study on the Effects of Salt Tolerance Type, Soil Salinity and Soil Characteristics on the Element Composition of Chenopodiaceae Halophytes [J]. Plants, 2022, 11(10): 1288.

[6] CHANG Y, FAN Y, LI Z, et al. Relationship between Photosynthetic Characteristics, Anatomical Structure, and Physiological Indexes of Two Halophytes in Different Habitats [J]. Forests, 2022, 13(12): 2189.

[7] LINHUI D. Allocation and Application of Saline-alkali Resistant Plants in Northern Coastal Areas [J]. Flowers, 2020, 2): 66-7.

[8] KORRENSALO A, ALEKSEYCHIK P, HáJEK T, et al. Species-specific temporal variation in photosynthesis as a moderator of peatland carbon sequestration [J]. Biogeosciences, 2017, 14(2): 257-69.

[9] YAN Z. Protective Countermeasures against Frost Damage to Landscape Plants in Tianjin Binhai New Area [J]. Journal of Green Science and Technology, 2010, 09): 57-8.

[10] SUN L, ZHANG J H J A M, MATERIALS. Multi-Angle to Solve the Problem of Urban Environment Greening-Several Suggestions of Urban Greening Based on Tianjin as Example [J]. Applied Mechanics, 2013, 361(514-8.

[11] HAEHLE R G, BROOKWELL J. Native Florida plants: Low maintenance landscaping and gardening [M]. Taylor Trade Publishing, 2004.

[12] HF Y, Y Z, WB X, et al. Negative impact assessment of urban green space ecological environment based on life cycle assessment [J]. Chinese Journal of Ecology 2023, 42(02): 493-503.

[13] ZHI-YI B, JIE-TING M. On the Design and Construction of Low-carbon Plant Landscape [J]. Chinese Landscape Architecture, 2011, 27(01): 7-10.

[14] RUIXUE L, YUANYUAN P. Evaluation of Plant Landscape in the Seaside Green Space Based on Analytic Hierarchy Process [J]. Journal of Northwest Forestry University, 2017, 32(4): 288-93.

[15] BELL S. Elements of visual design in the landscape [M]. Routledge, 2019.

[16] DANIEL T C J L, PLANNING U. Whither scenic beauty? Visual landscape quality assessment in the 21st century [J]. Landscape, 2001, 54(1-4): 267-81.

[17] CALLICOTT J B J E R. The land aesthetic [J]. Environmental review, 1983, 7(4): 345-58.

[18] WANG Z, LI M J F R M S. Methods of color plan of landscape forest at all seasons—a case study of Purple Mountain [J]. For Resour Manag S, 2017, 1.
